# Left ventricular remodeling in hypertrophic cardiomyopathy patients with atrial fibrillation

**DOI:** 10.1186/s12872-018-0945-7

**Published:** 2018-11-03

**Authors:** Hongwei Tian, Jingang Cui, Chengzhi Yang, Fenghuan Hu, Jiansong Yuan, Shengwen Liu, Weixian Yang, Xiaowei Jiang, Shubin Qiao

**Affiliations:** State Key Laboratory of Cardiovascular Disease, Fuwai Hospital, National Center for Cardiovascular Diseases, Chinese Academy of Medical Sciences and Peking Union Medical College, Beijing, 100037 China

**Keywords:** Atrial fibrillation, Hypertrophic cardiomyopathy, Left ventricular remodeling

## Abstract

**Background:**

Atrial fibrillation (AF) is the most common complication in hypertrophic cardiomyopathy (HCM). The mechanisms of AF is associated with left atrial (LA) structural remodeling in HCM patients. However, the impact of left ventricular (LV) remodeling on the presence of AF in HCM patients has not been evaluated yet. We sought to investigate effect of LV remodeling on the presence of AF assessed by cardiovascular magnetic resonance (CMR) in HCM patients.

**Methods:**

A total of 394 HCM patients were enrolled into this study, including HOCM patients (*n* = 293) and NOHCM patients (*n* = 101). Patients were divided into HCM with AF (50) and HCM without AF (*n* = 344). Data were collected from hospital records.

**Results:**

LA diameter and LV remodeling index (LVRI) were significantly higher in HCM patients with AF than that of HCM patients without AF (46.6 ± 7.4 mm versus 39.9 ± 8.0 mm, *p* < 0.001, and 1.46 ± 0.6 versus 1.2 ± 0.4, *p* = 0.002, respectively). HCM patients with AF were older than HCM patients without AF (53.6 ± 11.7 years versus 47.7 ± 13.6 years, *p* = 0.002). Additionally, LVRI positively correlated to LA size (*r* = 0.12, *p* = 0.02). In a multivariable logistic regression analysis, when adjusting for age and LV end diastolic mass index, LVRI and LA size remained an independent determinant of AF in HCM patients (OR = 4.7, *p* = 0.001 and OR = 1.13, *P* < 0.001).

**Conclusion:**

HCM patients with AF showed significantly more LA diameter, LVRI and age than HCM patients without AF. LVRI and LA size were strong independent predictor of AF in HCM, suggesting LV remodeling may contribute to the occurrence of AF in HCM patients.

## Background

Hypertrophic cardiomyopathy (HCM) is a complex and relatively common form of genetic heart disease characterized by left ventricular (LV) hypertrophy and the most frequent cause of sudden death in the young [1]. Histologically, HCM is characterized by myocyte disarray, scarring and microvascular dysfunction [2].

Atrial fibrillation (AF) is the most common arrhythmia in HCM and was associated with an increased risk for morbidity and mortality [3, 4]. The mechanisms of AF are complex and associated with structural and electrical remodeling in the atria and ventricular myocardium [5, 6]. In HCM patients, increased LA size, late gadolinium-enhancement (LGE) and advanced age have been shown to be independent predictors of the presence of AF [7, 2, 8]. However, the impact of LV remodeling on the presence of AF in HCM patients has not been evaluated yet. Thus, we used cardiovascular magnetic resonance (CMR) to evaluate effect of left ventricular remodeling index (LVRI) on the presence of AF in HCM patients.

## Methods

### Study population

**Fig. 1:**
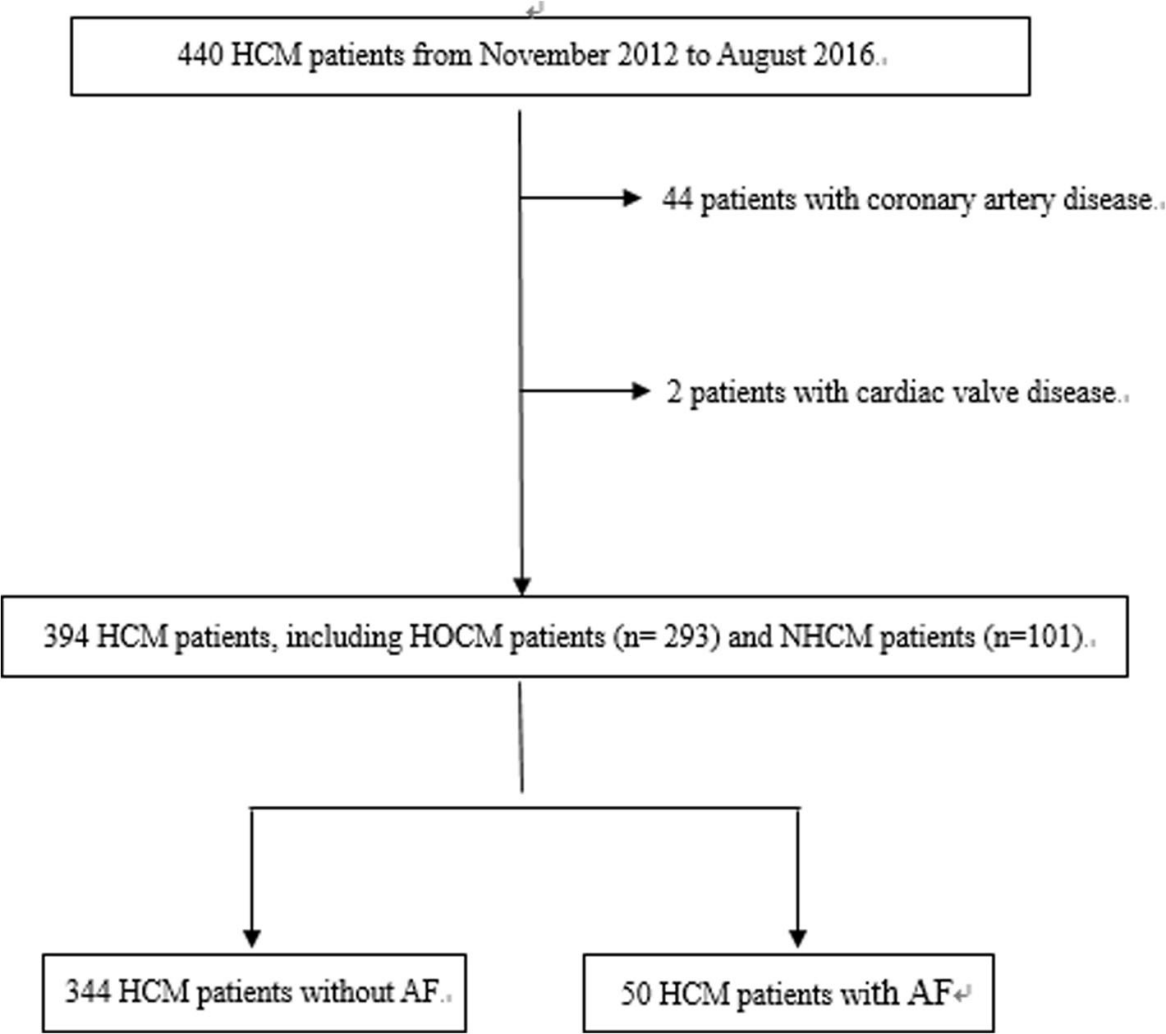
Flow chart of patient inclusion in the current study. LA, left atrial; HCM, hypertrophic cardiomyopathy; NOHCM, non-obstructive hypertrophic cardiomyopathy; HOCM, obstructive hypertrophic cardiomyopathy

The protocol study was approved by Fuwai Hospital ethics committee. The informed consents were obtained from all participants. We retrospectively analyzed data from 440 HCM patients who had maximum LV wall thickness ≥ 15 mm (or ≥ 13 mm with an unequivocal family history of HCM) in the absence of other cardiac or systemic causes of left ventricular hypertrophy [9, 10] between November 2012 and August 2016. Evaluation of patients included complete medical history, blood examination, physical examination, 24-h ambulatory electrocardiographic monitoring, transthoracic echocardiography, invasive coronary angiography, 12-lead electrocardiography and cardiovascular magnetic resonance imaging (MRI). Patients were excluded if they had (1) coronary artery disease (coronary artery stenosis > 50%), (2) renal dysfunction, (3) heart failure, (4) cardiac valve disease, (5) permanent mechanical device implantation. Forty-six patients were excluded owing to concomitant coronary artery disease (*n* = 44) and cardiac valve disease (*n* = 2) (Fig. 1). Finally, a total of 394 patients were enrolled into this study, including HOCM patients (*n* = 293) and NOHCM patients (*n* = 101). Patients were divided into HCM with AF (50) and HCM without AF (*n* = 344).

### Echocardiography

Standard transthoracic M-mode, 2-dimensional, and pulse-wave and continuous-wave Doppler images were obtained with an iE33 Color Doppler Ultrasound System (Philips Healthcare, Andover, Massachusetts). All measurements were analyzed following the guidelines of the American Society of Echocardiography. The left ventricular outflow tract (LVOT) peak gradient was estimated using the simplified Bernoulli eq. HCM with obstruction was defined as an LVOT peak gradient ≥30 mmHg at rest or provoked LVOT peak gradient > 50 mmHg. Patients were divided into non-obstructive (NOHCM) or obstructive (HOCM) based on left ventricular outflow tract obstruction [11].

### Cardiovascular MRI

**Fig. 2:**
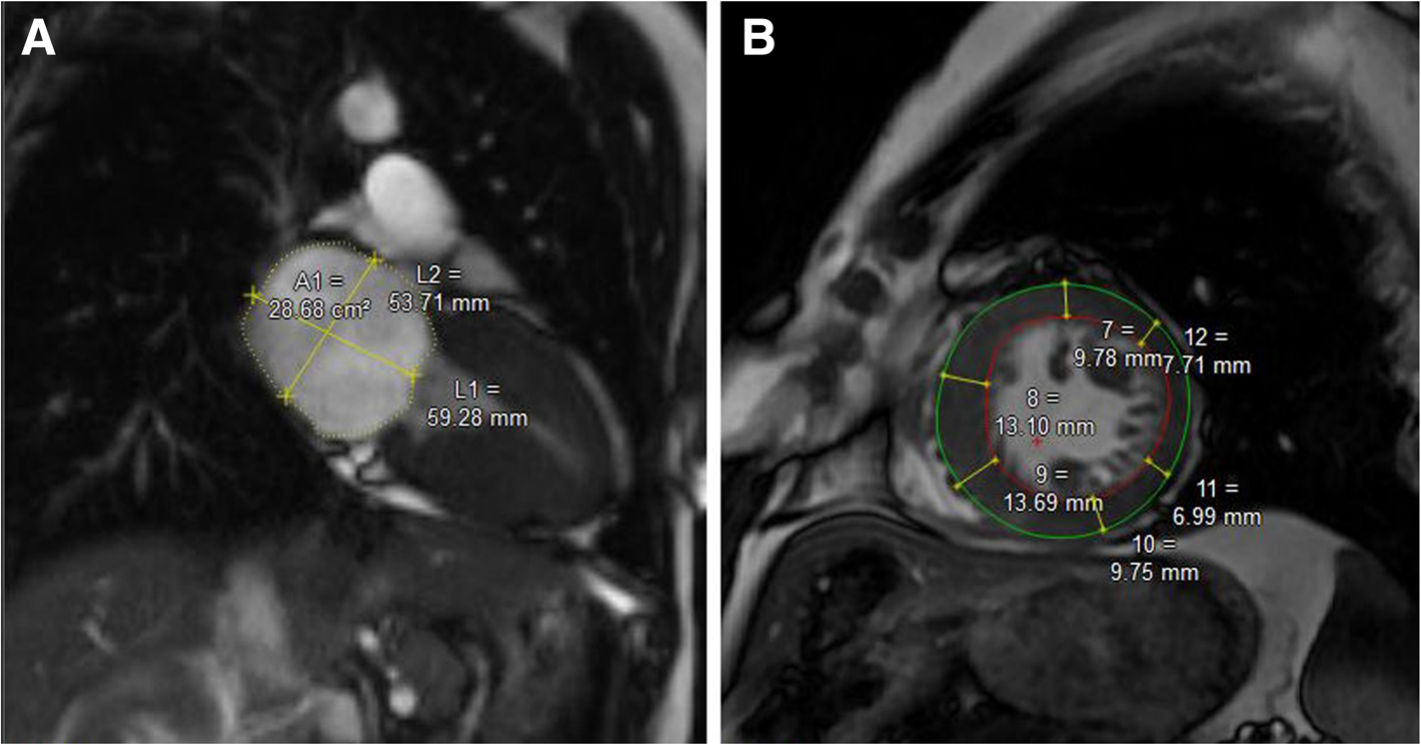
CMR images showed LA size (**a**) (yellow line) and contours of endocardial and epicardial (**b**) (red circle and green circle). CMR, cardiovascular magnetic resonance; LA, left atrial

CMR was performed using a 1.5-T speed clinical scanner (Magnetom Avanto; Siemens Medical Solutions, Erlangen, Germany). All MR image was analysed by a single experienced observer who was blinded to the all HCM patients. Endocardial contours of the LV myocardium were manually traced at end-diastole and end-systole on each LV short-axis cine image. LV end-diastolic volume (LVEDV), stroke volume, LV end-systolic volume (LVESV), LV ejection fraction (EF), and cardiac output were then calculated in a standard fashion. The LV end-diastolic diameter (EDD) was measured from short axis at LV end-diastolic phase and left atrial diameter (LAD) (Fig. 2a) was measured from transverse axis at LV end-systolic phase [11]. Left ventricular mass (LVM) was obtained on the basis of end-diastolic endocardial and epicardial contours (Fig. 2b) and calculated as the product of myocardial volume and specific density of myocardial tissue (1.05 g/ml). LVM and LV EDV were indexed to body surface area. Left ventricular remodeling index (LVRI = LVM/LV EDV) was calculated used the methods described previously [12].

### Atrial fibrillation

The diagnosis of AF was based on 12-lead electrocardiography or 24 h dynamic electrocardiogram recordings, or by an established history of paroxysmal or chronic AF [13].

### Statistical analysis

Statistical calculations were performed using SPSS 20.0 (SPSS Inc.; Chicago, IL, USA). In the case of a *p* < 0.05, the result was considered statistically significant. Data are expressed as mean ± SD for normally distributed continuous variables. Differences between means were measured by Student’s t-tests. Noncontinuous data were compared by chi-square tests as appropriate. Pearson correlation was used to evaluate the correlation between LA size and LVRI. Multivariate analysis was performed with logistic regression analysis using block entry of the following variables: LVRI, LA size, LV end diastolic mass index, and age to evaluate if these variables were independent predictors of AF, provided to have a *p* < 0.10 in univariate analysis.

## Results

### Patients characteristics

In our study, AF was documented in 50 HCM patients (12.7%). Baseline characteristics are presented in Table 1. No significant differences were observed for gender, systolic blood pressure (SBP), diastolic blood pressure (DBP), heart rate, NYHA class, body surface area (BSA), LVOT peak gradient. HCM patients with AF were older than HCM patients without AF (53.6 ± 11.7 years versus 47.7 ± 13.6 years, *p* = 0.002). The proportion of syncope, dyspnea, family history of HCM, family history of SCD, diabetes mellitus, hypertension, systolic anterior motion and use of medications did not differ significantly between HCM patients with AF and without AF groups.

**Table 1:** Patient Demographics and Baseline Characteristics

Variable	All Patients (*n* = 394)	Patients with AF (*n* = 50)	Patients without AF (*n* = 344)	*P* value
Age, y	48.5 ± 13.5	53.6 ± 11.7	47.7 ± 13.6	0.002
Male, n (%)	247 (62.7%)	37 (74%)	210 (61%)	0.08
Body surface area, m^2^	1.8 ± 0.2	1.8 ± 0.3	1.8 ± 0.2	0.41
NYHA class	2.4 ± 0.9	2.5 ± 0.9	2.4 ± 0.9	0.45
Heart rate, beats/min	70.5 ± 10.3	68.6 ± 9.9	70.8 ± 10.4	0.15
SBP (mmHg)	118.7 ± 17.2	120.0 ± 19.0	118.8 ± 15.7	0.63
DBP (mmHg)	73.0 ± 10.3	74.5 ± 10.9	72.7 ± 9.9	0.28
Syncope, n (%)	97 (24.6%)	13 (26%)	84 (24.4%)	0.81
Dyspnea, n (%)	319 (81%)	42 (84%)	277 (80.5%)	0.56
Hypertension, n (%)	116 (29.4%)	18 (36%)	98 (28.5%)	0.28
Diabetes mellitus, n (%)	13 (3.3%)	4 (8%)	9 (2.6%)	0.05
Family history of HCM, n (%)	56 (14.2%)	7 (14%)	49 (14.2%)	0.96
Family history of SCD, n (%)	24 (6.1%)	1 (2%)	23 (6.7%)	0.33
Medications, n (%)				
β-Blockers	261 (66.2%)	36 (72%)	225 (65.4%)	0.36
Echocardiography				
Systolic anterior motion	293 (74.4%)	36 (72%)	257 (74.7%)	0.68
LVOTPG at rest (mmHg)	74.9 ± 37.1	68.4 ± 46.9	75.9 ± 35.4	0.34

*HCM* hypertrophic cardiomyopathy, *LV* left ventricular, *LVOTG* LV outflow tract gradient, *NS* not significance; Values are expressed as either mean ± SD or number (percentage)

**Fig. 3:**
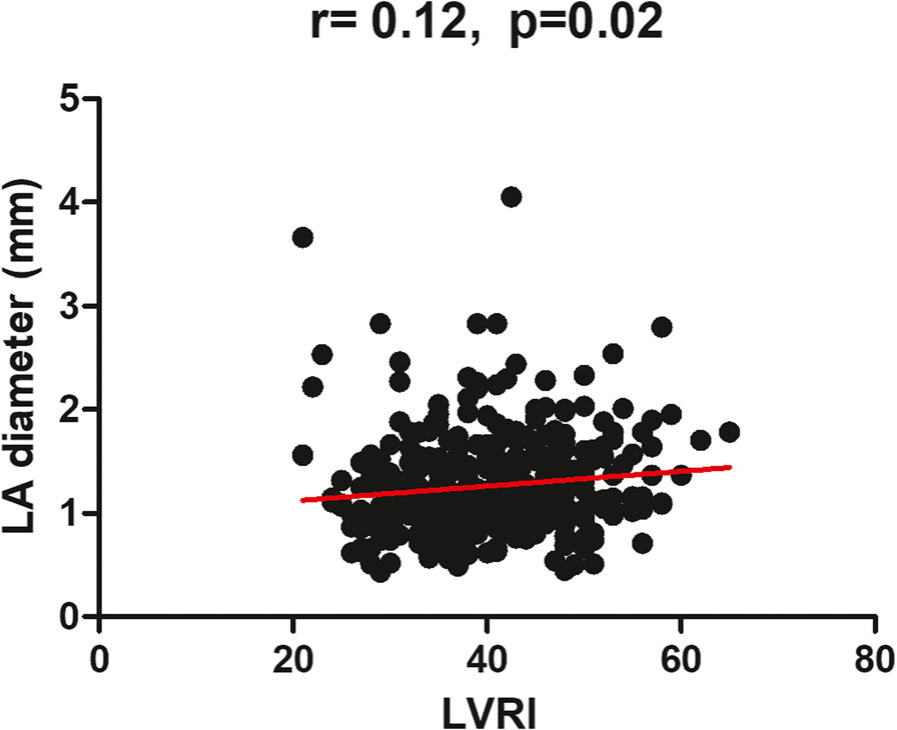
Scatterplots show significant correlations between LVRI and the LA size in all HCM patients. LVRI, left ventricular remodeling index; LA, left atrial; HCM, hypertrophic cardiomyopathy

LA and LV parameters, LV end diastolic mass index and LVRI were all comparable between HCM patients with AF and without AF, Table 2. Left atrial diameter and LVRI were significantly higher in HCM patients with AF than that of HCM patients without AF (46.6 ± 7.4 mm versus 39.9 ± 8.0 mm, *p* < 0.001, and 1.46 ± 0.6 versus 1.2 ± 0.4, *p* = 0.002). Additionally, pearson correlation analysis showed LVRI positively correlated to LA size (*r* = 0.12, *p* = 0.02) in all HCM patients, Fig. 3.

**Table 2:** CMR assessment

Variable	Patients with AF	Patients without AF	*P* value
LA dimension, mm	46.6 ± 7.4	39.9 ± 8.0	< 0.001
LVEDD, mm	47.0 ± 5.1	46.5 ± 5.3	0.48
LV ejection fraction, %	63.4 ± 12.3	67.2 ± 9.8	0.015
Septal wall thickness, mm	26.6 ± 4.5	24.7 ± 5.5	0.26
LV end diastolic volume index, ml/m^2^	69.6 ± 21.9	70.7 ± 16.7	0.72
LV ESVI	26.3 ± 14.5	23.7 ± 11.4	0.22
CI	3.0 ± 1.0	3.2 ± 0.8	0.14
LV end diastolic mass index, g/m^2^	95.8 ± 36.7	85.7 ± 34.8	0.07
LVRI	1.46 ± 0.6	1.2 ± 0.4	0.002

Data are presented as ± standard deviation. Volumes are indexed to body surface area. *EDD* end diastolic dimension, *LA* left atrial, *LV* left ventricular, *ESVI* end-systolic volume index, *CI* Cardiac index

In HOCM patients, LA dimension (*p* = 0.025), LV ejection fraction (*p* < 0.001), septal wall thickness (*p* < 0.001), LV end diastolic mass index (*p* < 0.001) and LVRI (*p* < 0.001) were significantly larger and LV EDD (*p* < 0.001) was lower compared to NOHCM patients. However, there was no significant differences between HOCM patients and NOHCM patients regarding the occurrence of AF (12.6% vs. 12.9%, *p* = 0.95), see Table 3.

**Table 3:** Comparison of left ventricular and left atrial dimensions between HOCM and NOHCM patients

Variable	HOCM	NOHCM	*P* value
AF, %	12.6%	12.9%	0.95
LA dimension, mm	40.4 ± 8.5	38.4 ± 7.7	0.025
LVEDD, mm	46.0 ± 5.2	48.3 ± 5.0	< 0.001
LV ejection fraction, %	68.5 ± 9.2	61.7 ± 11.2	< 0.001
Septal wall thickness, mm	23.6 ± 5.4	21.0 ± 6.3	< 0.001
LV end diastolic volume index, ml/m^2^	71.4 ± 18.6	68.2 ± 13.2	0.12
LV ESVI, ml/m^2^	23.1 ± 11.7	26.8 ± 11.9	0.008
CI, ml/m^2^	3.3 ± 0.9	2.8 ± 0.7	< 0.001
LV end diastolic mass index, g/m^2^	91.4 ± 35.5	74.4 ± 31.0	< 0.001
LVRI	1.3 ± 0.5	1.1 ± 0.5	< 0.001

*AF* atrial fibrillation, *LA* left atrial, *EDD* end diastolic dimension, *LV* left ventricular, *ESVI* end-systolic volume index, *CI* Cardiac index, *LVRI* left ventricular remodeling index

In a multivariable logistic regression analysis, when adjusting for age and LV end diastolic mass index, LVRI and LA size remained an independent determinant of AF in HCM patients (OR = 4.7, *p* = 0.001 and OR = 1.13, *P* < 0.001), see Table 4.

**Table 4:** Predictors of AF in HCM group by univariate and multivariable logistic regression

Variable	Univariate analysis			Multivariable logistic regression		
	*P* value	Crude OR	95% CI	*P* value	Adjusted OR	95% CI
Age	0.004	1.0	1.0~ 1.1	0.01	1.0	1.0~ 1.1
LA dimension, mm	< 0.001	1.1	1.1~ 1.2	< 0.001	1.13	1.1~ 1.2
LV end diastolic mass index, g/m^2^	0.06	1.0	1.0~ 1.02	0.07	0.99	0.97~ 1.0
LVRI	0.003	2.3	1.3~ 4.0	0.001	4.7	1.9~ 11.8

*LA* left atrial, *LV* left ventricular, *LVRI* left ventricular remodeling index

## Discussion

The present study demonstrates that HCM patients with AF had higher LA diameter, age and LVRI than HCM patients without AF. LA size mildly correlated to LVRI in all HCM patients. When adjusting for age and LV end diastolic mass index, LVRI and LA size remained an independent determinant of AF in HCM patients.

AF is a commonly reported complication in HCM that affects quality of life and increases risk for morbidity and mortality. It has been previously revealed that the diagnosis of HCM precedes the presence of AF in the majority of HCM patients [3] which strongly suggests that the structural and physiological changes related to the development of AF. In HCM patients, diastolic dysfunction, advanced age, myocardial ischemia, myocardial fibrosis, LA diameter and congestive heart failure symptoms have been shown to be associated with the development of AF [7, 14]. However, the impact of LV remodeling on the presence of AF in HCM patients has not been evaluated yet. The aim of the present study was to investigate whether LV remodeling is related to the occurrence of AF in HCM patients.

LA dimension is one of the most important determinants of AF occurrence in HCM patients. In our study, we showed that LA diameter and age was significantly higher in HCM patients with AF than that of HCM patients without AF, these findings confirm previous study [2, 13, 15]. In the present study, we also showed LVRI positively correlated to LA size, suggesting that LV remodeling may contribute to the enlargement of LA. LA enlargement is a multifactorial process in HCM, including LA overload, mitral regurgitation, intrinsic myocardial stiffness, LV diastolic dysfunction and rhythm disturbances [14, 16, 17].

The LVRI which was calculated as the ratio of LV mass and end-diastolic volume can evaluate the degree of LV remodeling [6]. In our study, HCM patients with AF had higher LV mass index and LVRI. In a multivariable logistic regression analysis, LVRI and LA size remained an independent determinant of AF in HCM patients. These observations indicate that LA size and progressive LV remodeling may contribute to the occurrence of AF in HCM patient. The main underlying structural abnormalities in HCM include myocardial cell disarray, coronary microvasculature dysfunction and remodeling changes [18, 19]. LV myocardial remodeling that occur as a compensatory mechanism and can involve changes to the fibroblasts, myocytes and interstitium. LV remodeling and increased LV mass impaired diastolic function due to increased myocardial stiffness and decreased chamber compliance [17]. Moreover, LV diastolic dysfunction can lead to LA enlargement and associated rhythm disturbances [20]. Patients with AF frequently have the left atrial appendage remodeling in which there is dilation, stretching, and reduction in pectinate muscle volume [21]. Prior studies have showed that LA diameter and P wave dispersion values are the most significant predictors for AF occurrence in patients with HCM [22]. All these findings suggested that the AF was a result of electrical remodeling and myocardial remodeling [23]**.**

### Limitations

There may be some limitations in our study. Firstly, we did not evaluate the impact of late gadolinium enhanced (LGE) on the presence of AF in HCM patients owning to the absence of LGE examination. Secondly, in this study, patients with hypertension were not excluded.

## Conclusions

HCM patients with AF showed significantly more LA diameter, LVRI and age than HCM patients without AF. LVRI and LA size were strong independent predictor of AF in HCM, suggesting that the LA enlargement and progressive LV remodeling may contribute to the occurrence of AF in HCM patients.

## Abbreviations

AF: Atrial fibrillation
CMR: Cardiovascular magnetic resonance
DBP: Diastolic blood pressure
EDD: End-diastolic diameter
EF: Ejection fraction
HCM: Hypertrophic cardiomyopathy
HOCM: Obstructive hypertrophic cardiomyopathy
LA: left atrial
LAD: Left atrial diameter
LGE: Late gadolinium-enhancement
LV: Left ventricular
LVEDV: LV end-diastolic volume
LVESV: LV end-systolic volume
LVM: Left ventricular mass
LVOT: Left ventricular outflow tract
LVRI: LV remodeling index
NOHCM: Non-obstructive hypertrophic cardiomyopathy
SBP: systolic blood pressure
